# Non-Uniform Entropy-Constrained *L*_∞_ Quantization for Sparse and Irregular Sources

**DOI:** 10.3390/e27111126

**Published:** 2025-10-31

**Authors:** Alin-Adrian Alecu, Mohammad Ali Tahouri, Adrian Munteanu, Bujor Păvăloiu

**Affiliations:** 1 Faculty of Engineering in Foreign Languages (FILS), Universitatea Nationala de Stiinta si Tehnologie Politehnica Bucuresti, Splaiul Independentei 313, 060042 Bucharest, Romania; bujor.pavaloiu@upb.ro; 2 Department of Electronics and Informatics (ETRO), Vrije Universiteit Brussel, Pleinlaan 2, 1050 Brussels, Belgium; mohammad.ali.tahouri@vub.be (M.A.T.); adrian.munteanu@vub.be (A.M.)

**Keywords:** near-lossless compression, *L*_∞_ quantization, non-uniform scalar quantizers, sparse distributions, depth map coding

## Abstract

Near-lossless coding schemes traditionally rely on uniform quantization to control the maximum absolute error ($L_{\infty }$ norm) of residual signals, often assuming a parametric model for the source distribution. This paper introduces a novel design framework for non-uniform, entropy-aware $L_{\infty }$-oriented scalar quantizers that leverages a tight and differentiable approximation of the $L_{\infty }$ distortion metric and does not require any parametric density function formulations. The framework is evaluated on both synthetic parametric sources and real-world medical depth map video datasets. For smoothly decaying distributions, such as the continuous Laplacian or discrete two-sided geometric distributions, the proposed method naturally converges to near-uniform quantizers, consistent with theoretical expectations. In contrast, for sparse or irregular sources, the algorithm produces highly non-uniform bin allocations that adapt to the local distribution structure and improve rate-distortion efficiency. When embedded in a residual-based near-lossless compression scheme, the resulting codec consistently outperforms versions equipped with uniform or piecewise-uniform quantizers, as well as state-of-the-art near-lossless schemes such as JPEG-LS and CALIC.

## 1. Introduction

Advances in data acquisition technologies have led to an ever-increasing variety of digital media that require efficient storage and transmission, ranging from natural, medical, and satellite images to sensor data such as depth maps or point clouds. For such data, near-lossless compression has emerged as an attractive paradigm, offering a controlled trade-off between bitrate reduction and fidelity preservation. Lossless compression reproduces the original data exactly but achieves limited compression efficiency, whereas lossy compression attains higher rates by allowing average distortions, typically measured under the $L_{2}$ norm. Near-lossless compression lies between these two extremes, ensuring that each reconstructed sample ${\hat{x}}_{i}$ deviates from the original $x_{i}$ by at most a specified maximum absolute error $D_{\max}$, i.e., $|x_{i}-{\hat{x}}_{i}|\leq D_{\max}$ for all *i*, which corresponds to a bounded $L_{\infty }$ distortion. This feature is particularly important for applications where local accuracy is critical, such as medical imaging or 3D reconstruction from depth maps.

Existing near-lossless coding schemes have been developed using a variety of approaches, including spatial-domain coding, predictive coding, transform coding, progressive coding, and, more recently, deep learning-based solutions. Despite their effectiveness, most methods employ uniform quantizers designed under specific source distribution assumptions. Residual distributions are commonly modeled using continuous Gaussian-like distributions or discrete families such as the two-sided geometric distribution (TSGD), which justifies the use of uniform quantizers that are theoretically $L_{\infty }$-optimal in these cases. Overall, although quantizer design is well understood for classical average-error criteria such as the $L_{2}$ metric, extending these designs to entropy-constrained $L_{\infty }$ quantizers remains largely distribution-dependent. In practice, near-lossless schemes continue to rely on uniform quantizers, whose $L_{\infty }$ optimality is generally limited to symmetric, unimodal, and monotone residual distributions. Indeed, classical quantization theory shows that uniform scalar quantizers are near-optimal primarily for smooth, symmetric, and unimodal distributions that decrease monotonically from their mode [1,2]. For asymmetric or multimodal sources, the equal spacing of decision thresholds causes certain regions to be over- or under-represented, leading to inefficient bit allocation and unbalanced reconstruction errors. This observation emphasizes the need for quantization schemes capable of efficiently handling sparse or otherwise non-standard sources.

To overcome this limitation, we introduce a general framework for entropy-constrained scalar quantizer design under the $L_{\infty }$ distortion metric, capable of adapting to arbitrary, possibly sparse or non-Gaussian source distributions. The framework relies on a differentiable approximation of the $L_{\infty }$ norm and operates directly on input signal samples, without requiring an explicit analytical form of the source probability density function $f_{X}\left(x\right)$. It produces as output an optimized set of decision boundaries and reconstruction levels obtained by minimizing a rate–distortion Lagrangian that can be adjusted to satisfy either a target distortion bound $D_{\max}$ or a target rate $R_{\max}$, providing flexible control of compression behavior without parametric modeling or prior assumptions. We show that for Gaussian-alike continuous distributions and for the TSGD case, our algorithm converges to uniform quantizers, thereby confirming the $L_{\infty }$-optimality assumptions widely used in the literature. In contrast, for discontinuous or sparse distributions, the scheme produces non-uniform quantizers that adapt to the structure of the source and clearly outperform uniform quantizers in the $L_{\infty }$ rate–distortion sense.

The proposed framework is suited for applications that require strict local fidelity and involve sparse or irregular data, such as 3D sensing, robotics, remote sensing, and medical imaging. By adapting the quantization intervals to source statistics while maintaining a guaranteed maximum error bound, it enables efficient near-lossless compression across these domains.

To demonstrate the practical benefits, we integrate the proposed quantizers into a residual-based near-lossless encoder for depth video sequences acquired in medical and assistive monitoring contexts. Experimental results show that this codec outperforms both its uniform-quantizer counterparts and state-of-the-art near-lossless compression schemes such as JPEG-LS and CALIC.

The main contributions of this paper are summarized as follows:

- We propose an iterative scheme for entropy-constrained scalar $L_{\infty }$-oriented non-uniform quantizers that is applicable to sparse (discontinuous) input distributions.
- We demonstrate that the algorithm converges to uniform designs for smooth symmetric sources commonly used to model residuals and yields non-uniform quantizers for sparse or irregular distributions.
- We embed the proposed quantization scheme into a residual-based near-lossless depth video codec and show that it consistently outperforms state-of-the-art methods such as JPEG-LS and CALIC.

The remainder of this paper is organized as follows. Section 2 reviews related work on quantizer design under the $L_{\infty }$ metric and on near-lossless compression. Section 3 introduces the proposed entropy-constrained $L_{\infty }$-oriented quantizer design algorithm. Section 4 presents experimental results, including both synthetic distribution tests and depth video coding. Section 5 provides a discussion of the findings, and Section 6 concludes this paper.

## 2. Related Work

This section reviews prior work in three areas: quantizer design under general $L_{p}$ metrics, with emphasis on the $L_{\infty }$ norm; near-lossless compression of natural images and video; and near-lossless compression of depth maps, highlighting methods that provide $L_{\infty }$ guarantees.

### 2.1. Optimal Quantizer Design for $L_{p}$ and $L_{\infty }$ Distortion

The problem of optimal quantizer design has been extensively studied in the context of various distortion measures. The early work of Lloyd and Max [3,4] introduced an iterative design of non-uniform PDF-optimized scalar quantizers for fixed-length coding using the $L_{2}$ distortion metric, which was later extended to vector quantization by Linde, Buzo, and Gray as the generalized Lloyd algorithm [5]. In variable-length coding, Woods [6] introduced the first numerical descent algorithm for entropy-constrained scalar quantizers, while Berger [7] proposed a Lagrangian formulation of the optimization problem and derived optimality conditions for entropy-constrained scalar quantizers operating under the $L_{2}$ distortion metric. Farvardin and Modestino [8] further extended these results by adapting the framework to a broader class of distortion measures, while Chou et al. [9] generalized the work of [7,8] to entropy-constrained vector quantization and derived optimal conditions for minimizing the Lagrangian function. Zamir and Feder later showed that dithered uniform and lattice quantizers achieve near-optimal rate–distortion performance, defining a universal framework for entropy-constrained design [10]. General references on quantization theory are given by Gersho and Gray [11] and Gray and Neuhoff [2].

When discussing optimal quantizer design for a given distortion measure, several works [5,8] consider a general class of non-negative distortion measures, including specific instances *p* of the broader Hölder distortion $L_{p}$, and show that the optimization framework applies across these measures. While many studies provide explicit solutions for both fixed-rate and variable-rate quantization under the $L_{2}$ distortion metric, designing quantizers for the $L_{\infty }$ measure is more challenging due to the non-differentiability of the maximum-error criterion. Mathews and Hahn [12] address this by considering the limiting case p→∞ of the $L_{p}$ norm and propose an iterative algorithm for fixed-rate vector quantization under the $L_{\infty }$ metric; however, extending their approach to entropy-constrained quantization is not straightforward. Linder and Zamir further showed that optimality conditions remain valid for general distortion measures beyond $L_{2}$, including bounded-error criteria [13].

More recently, Ling and Li proposed a rejection-sampled universal quantizer that minimizes the maximum reconstruction error under an entropy constraint [14].

At the distributional level, Chang et al. [15] modeled depth residuals using a TSGD and showed that uniform scalar quantizers are $L_{\infty }$-optimal for this distribution, providing a distribution-dependent solution specific to TSGD sources. Schiopu and Tabus observed similar bounded-error efficiency for Laplacian and exponential residuals in near-lossless depth coding [16]. More generally, uniform quantizers are considered $L_{\infty }$-optimal for symmetric, unimodal, piecewise monotone distributions, such as Gaussian and Laplacian residuals [11]. These approaches, however, remain tied to specific distributional assumptions and do not generalize to arbitrary sparse or discontinuous source distributions.

### 2.2. Near-Lossless $L_{\infty }$-Oriented Compression Schemes

Near-lossless image and video compression has been explored through a variety of paradigms, each leveraging different strategies to bound the reconstruction error while maintaining coding efficiency.

*Spatial domain coding* techniques operate directly on pixel values, often exploiting local correlations. The early work of Chen and Ramabadran [17] proposed differential pulse-code modulation (DPCM) coding combined with uniform quantization to ensure that the $L_{\infty }$ distortion does not exceed a predefined bound of 1. This approach was later generalized by Ke and Marcellin [18] to support arbitrary discrete distortion constraints. A modern implementation of near-lossless spatial coding is found in the WebP standard [19], which preprocesses pixel values to reduce local differences, effectively enforcing an $L_{\infty }$ error bound while maintaining compression efficiency suitable for web applications.

*Predictive coding* relies on estimating pixel values from previously encoded pixels and then encoding only the residual error. Avcıbaş and Memon [20] proposed a progressive predictive scheme with uniform quantization of residuals, enabling near-lossless $L_{\infty }$ compression with scalable refinement. Wu and Bao [21] extended the CALIC lossless compression scheme [22] to the near-lossless setting, using uniform quantization to enforce an $L_{\infty }$ error constraint. JPEG-LS [23], a widely adopted standard, combines predictive modeling with uniform quantization of the prediction residuals, allowing for effective $L_{\infty }$ control. More recently, Tahouri et al. [24] proposed a lightweight codec for depth video that operates in both lossless and near-lossless mode, using uniform quantization to set bounds on the $L_{\infty }$ error.

*Transform coding* approaches first decorrelate image data using a transform, such as a filterbank or wavelet, and then quantize the transform coefficients. Karray et al. [25] formulated $L_{\infty }$-constrained coding in a probabilistic framework using filterbanks to manage coefficient quantization errors. Ansari et al. [26] proposed a hybrid method that combines predictive and transform coding, enhancing coding efficiency under near-lossless constraints. Alecu et al. [27] introduced a scalable $L_{\infty }$ coding scheme based on the lifting wavelet transform, enabling multi-resolution compression with per-pixel error bounds.

*Progressive or embedded coding* allows for embedded bitstreams that can be truncated for lossy-to-lossless decoding. Pinho and Neves [28] introduced a progressive lossless compression method optimized for $L_{\infty }$-constrained decoding.

*Deep learning*-based approaches have also been explored for near-lossless compression. Zhang and Wu [29] applied convolutional neural networks (CNNs) within a generative adversarial framework to improve reconstruction quality while respecting $L_{\infty }$ constraints. Zhang et al. [30] developed a CNN-based near-lossless codec using uniform quantization of latent features. Bai et al. [31,32] proposed variational autoencoder architectures for joint lossy and residual coding, achieving near-lossless performance by combining learned representations with uniform quantization of residuals to satisfy a prescribed $L_{\infty }$ error bound.

### 2.3. Near-Lossless $L_{\infty }$ Compression of Depth Maps

While the previous subsection focused on near-lossless compression of natural images and video, depth map data presents unique challenges due to its sparsity and wide dynamic range. Several codecs have been proposed for near-lossless depth compression. Mehrotra et al. [33] introduced a low-complexity inverse coding scheme that combines prediction and adaptive Run-Length Golomb–Rice coding. Choi and Ho [34] enhanced HEVC’s near-lossless mode for depth sequences by performing statistical analysis of residual data. Shahriyar et al. [35] presented a depth sequence coder using hierarchical partitioning and spatial-domain uniform quantization. Von Bülow et al. [36] applied depth-of-field segmentation to achieve near-lossless compression, effectively prioritizing perceptually important regions of depth data.

Recent approaches include Siekkinen [37], who proposed neural network-assisted packing of depth maps into video frames; the method reduces compression errors and can support near-lossless operation. Wu and Gao [38] presented an end-to-end lossless compression method for high-precision depth maps, which can be adapted for near-lossless use by allowing small controlled errors.

While all these approaches are near-lossless in nature, only a few explicitly enforce $L_{\infty }$-bounded coding of depth maps. Notably, Chang et al. [15] introduced an $L_{\infty }$-predictive coding scheme for depth sequences, while Tahouri et al. [24] developed a lossless and near-lossless codec tailored to depth video data with strict $L_{\infty }$ error guarantees.

## 3. Materials and Methods

In this section, we propose a quantizer design scheme for entropy-constrained $L_{\infty }$-oriented scalar quantizers. A key challenge is that the standard definition ${∥X∥}_{\infty }=\max_{i}\left|x_{i}\right|$ is piecewise and non-smooth. Its gradient is sparse (depending only on the component attaining the maximum) and becomes undefined at points where the maximum switches between components $x_{i}$. To enable stable optimization, we introduce a tight differentiable approximation of the $L_{\infty }$ quantization error, which we then use to formulate the Lagrangian optimization problem for entropy-constrained scalar quantizers. Solving this problem yields optimality conditions that form the basis of our iterative $L_{\infty }$-oriented quantizer design algorithm.

### 3.1. A Differentiable Approximation of the $L_{\infty }$ Quantization Error

We model a discrete input source as a random field of *N* i.i.d. random variables $X_{1},\ldots X_{N}$ with a common density function $f_{X}\left(x\right)$, respectively, as a single random variable *X* with $f_{X}\left(x\right)$. This formulation adopts the i.i.d. source model as a standard simplifying assumption in quantization and entropy–distortion theory [2,11], allowing the analysis to focus on the marginal distribution of the source rather than on its joint dependencies. Although real-world data may exhibit statistical correlations, the derivation of scalar quantizer properties and entropy–constrained bounds relies only on the marginal behavior of *X*, which sufficiently characterizes local amplitude statistics under the memoryless model. Given a quantization operation Q(·), we denote by $\left\{b_{i}\right\}$ and $\left\{y_{i}\right\}$ the decision boundaries and reconstruction levels associated with its quantization intervals. The following theorem provides a differentiable approximation of the $L_{\infty }$ quantization error.

**Theorem** **1.** *Let X be a random variable with probability density function $f_{X}\left(x\right)$, and consider a scalar quantizer Q(·) defined by decision boundaries ${\left\{b_{i}\right\}}_{i=0}^{M+1}$ and reconstruction levels ${\left\{y_{i}\right\}}_{i=0}^{M}$, such that $Q\left(x\right)=y_{i}$ for any $b_{i}\leq x<b_{i+1}$. Then, the expected $L_{\infty }$ quantization error $d\left(X,Q\left(X\right)\right)=\max_{i}\left|x_{i}-Q\left(x_{i}\right)\right|$ can be approximated as follows, where τ>0 is a parameter that controls the tightness of the approximation:*

(1) $$E\left[d\left(X,Q\left(X\right)\right)\right]\approx \frac{1}{\tau }\log\sum_{i=0}^{M}\int_{b_{i}}^{bi+1}e^{\tau \left|x-y_{i}\right|}f_{X}\left(x\right)dx,$$

A concise proof is provided below, while the full derivation is given in Appendix A.

**Proof** **of** **Theorem** **1.** Let us define $U=\tau \max_{j=1\ldots N}\left|Z_{j}\right|$ with some parameter τ>0 and where $Z_{j}=X_{j}-Q\left(X_{j}\right)$. By Jensen’s inequality φ(E[U])≤E[φ(U)] for a convex function φ, it follows that:

(2) $$e^{\tau E\left[\max_{j}\left|Z_{j}\right|\right]}\leq E\left[e^{\tau \max_{j}\left|Z_{j}\right|}\right]\leq E\left[\sum_{j=1}^{N}e^{\tau |Z_{j}|}\right]\leq NE\left[e^{\tau \max_{j}\left|Z_{j}\right|}\right],$$

Taking the logarithm in (2) and using Jensen again, we obtain:

(3) $$E\left[\max_{j}\left|Z_{j}\right|\right]\leq \frac{1}{\tau }\log E\left[\sum_{j=1}^{N}e^{\tau |Z_{j}|}\right]\leq E\left[\max_{j}\left|Z_{j}\right|\right]+\frac{\log N}{\tau },$$

The middle term can be written in terms of a single random variable as $E\left[\sum_{j=1}^{N}e^{\tau |Z_{j}|}\right]=NE\left[e^{\tau |Z|}\right]$, such that (3) leads to:

(4) $$\frac{1}{\tau }\log E\left[e^{\tau |Z|}\right]\leq E\left[\max_{j}\left|Z_{j}\right|\right]\leq \frac{1}{\tau }\log E\left[e^{\tau |Z|}\right]+\frac{\log N}{\tau },$$

Equation (4) gives a tight approximation $\frac{1}{\tau }\log E[e^{\tau }\left|Z|\right]$ for $E[\max_{j}|Z_{j}|]$. Since $E\left[d(X,Q(X))\right]=\int_{-\infty }^{\infty }d\left(x,Q\left(x\right)\right)f_{X}\left(x\right)dx$ can be written as $\sum_{i=0}^{M}\int_{b_{i}}^{bi+1}d\left(x,y_{i}\right)f_{X}\left(x\right)dx$, Equation (1) easily follows, which concludes the proof. □

Finally, it is interesting to note that Equation (1) is derived using the bounds (3) of the LogSumExp function $\log\sum_{j=1}^{N}e^{\tau |Z_{j}|}$, which is a well-known smooth approximation of the max(·) function. Furthermore, with the exception of discontinuities introduced by the |·| operator, the $L_{\infty }$ quantization error (1) is now differentiable.

To clarify the practical meaning of the above result, we present a simple numerical example comparing the theoretical bound of Equation (1) with its empirical evaluation.

**Numerical Illustration of the Theorem.** Let τ=0.2. We consider a scalar quantizer defined by decision boundaries $\left\{b_{i}\right\}$ and reconstruction points $\left\{y_{i}\right\}$ derived from a Laplacian dataset with σ=10. The quantizer is fixed, and multiple independent datasets ${\left\{x_{j}^{\left(k\right)}\right\}}_{j=1}^{N}$, $k=1,\ldots ,10^{4}$, are generated from the same distribution. For each dataset, the maximum absolute quantization error $D^{\left(k\right)}=\max_{j=1:N}\left|x_{j}^{\left(k\right)}-Q\left(x_{j}^{\left(k\right)}\right)\right|$ is computed, yielding an empirical distribution of maxima. We calculate the empirical mean $E[D^{\left(k\right)}]$ that corresponds to the left-hand side of (1), the theoretical value of the right-hand side integral for the Laplacian density function and the given quantizer, and the analytical upper bound obtained by adding the bound $\frac{\log N}{\tau }$.

**Figure 1:**
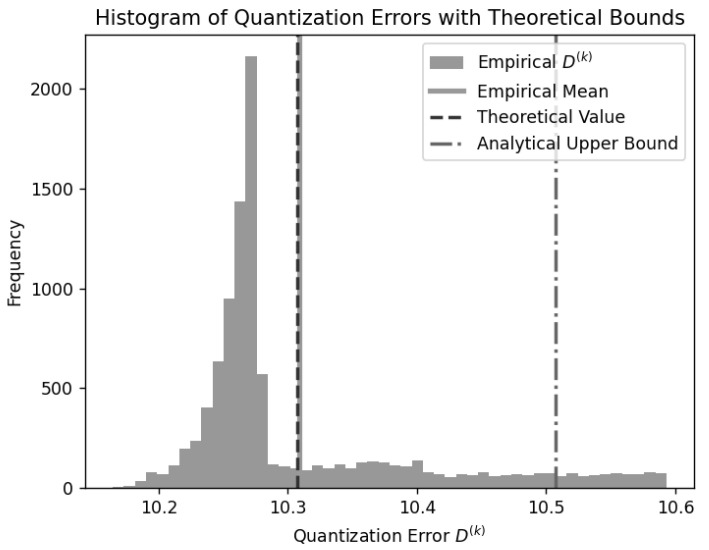
Empirical and theoretical $L_{\infty }$ quantization errors for a Laplacian source.

Figure 1 shows the histogram of empirical maxima, with vertical lines marking the empirical mean, theoretical value, and analytical upper bound. As each $D^{\left(k\right)}$ represents the maximum quantization error over many samples, its distribution follows extreme value statistics and approaches a Gumbel form as *N* increases. The theoretical value slightly underestimates the empirical mean but remains remarkably close to it, confirming the accuracy of the approximation. Both values lie well below the analytical upper bound, which provides a conservative margin consistent with the theoretical inequality.

### 3.2. An Iterative Quantizer Design Algorithm

The design of optimal fixed-rate quantizers can be formulated as finding a quantizer Q(·) that minimizes the distortion D(Q) subject to a rate constraint $R\left(Q\right)\leq R_{\max}$, or, in the dual approach, as minimizing the rate R(Q) subject to a maximum allowable distortion $D\left(Q\right)\leq D_{\max}$. In what follows, we focus on the classical first formulation, noting that the second case follows in a similar manner.

Using the distortion expression of (1), a Lagrangian formulation for $L_{\infty }$-oriented entropy-constrained scalar quantization can be written as:

(5) $$J=\frac{1}{\tau }\log\sum_{i=0}^{M}\int_{b_{i}}^{b_{i+1}}e^{\tau |x-y_{i}|}f_{X}\left(x\right)dx+\lambda \sum_{i=0}^{M}l_{i}\int_{b_{i}}^{b_{i+1}}f_{X}\left(x\right)dx,$$

where λ is a Lagrangian multiplier and $l_{i}$ are codeword lengths.

To simplify expressions, let us first denote:

(6) $$\Phi =\sum_{i=0}^{M}\int_{b_{i}}^{b_{i+1}}e^{\tau |x-y_{i}|}f_{X}\left(x\right)dx,$$

The optimal reconstruction levels ${\left\{y_{i}\right\}}_{i=0}^{M}$ and decision levels ${\left\{b_{i}\right\}}_{i=0}^{M+1}$ that minimize (5) can be found by computing derivatives. For readability, we present only the main steps; full intermediate derivations for this subsection are provided in Appendix A. For high rates, we assume that the density function $f_{X}\left(x\right)$ is constant over each quantization interval $\left[b_{i},b_{i+1}\right)$, i.e., has the value $f_{X}\left(y_{i}\right)$, such that $\frac{dJ}{dy_{i}}$ becomes:

(7) $$\frac{dJ}{dy_{i}}=\frac{f_{X}\left(y_{i}\right)}{\tau \Phi }\frac{d}{dy_{i}}\left(\int_{y_{i}}^{b_{i+1}}e^{\tau (x-y_{i})}dx+\int_{b_{i}}^{y_{i}}e^{\tau (y_{i}-x)}\right)dx,$$

Further computing (7) and setting it to zero gives us:

(8) $$e^{\tau (y_{i}-b_{i})}-e^{\tau (b_{i+1}-y_{i})}=0,$$

From (8), we now obtain the reconstruction level condition:

(9) $$y_{i}=\frac{b_{i}+b_{i+1}}{2},$$

We now compute $\frac{dJ}{db_{i}}$ by applying the Leibniz integral rule for (5), leading to the following expression:

(10) $$\frac{dJ}{db_{i}}=f_{X}\left(b_{i}\right)\left(\frac{e^{\tau (b_{i}-y_{i-1})}-e^{\tau (y_{i}-b_{i})}}{\tau \Phi }-\lambda \left(l_{i}-l_{i-1}\right)\right),$$

Setting (10) to zero and substituting the expression from (6) would, in principle, allow the calculation of the optimal values of $b_{i}$. However, this approach does not yield analytical solutions for $b_{i}$, though they can be approximated using numerical methods. Consequently, we propose an alternative method that provides a near-optimal solution.

Specifically, from (5) and (6), we have $D=\frac{1}{\tau }\log\Phi$, where *D* is the $L_{\infty }$ distortion. It is evident upon inspection that most bin decision levels $b_{i}$ have a negligible effect on *D* (and hence on Φ), except for the few bins responsible for the maximum quantization error. This behavior is analogous to the LogSumExp function, where exponential terms ensure that only the largest contributions dominate. Based on this observation, we assume that Φ is effectively independent of $b_{i}$ for the majority of bins. Under this assumption, setting (10) to zero leads to the following equation for $e^{\tau b_{i}}$:

(11) $$e^{2\tau b_{i}}-\tau \Phi \lambda \left(l_{i}-l_{i-1}\right)e^{\tau y_{i-1}}e^{\tau b_{i}}-e^{\tau (y_{i-1}+y_{i})}=0,$$

Expression (11) can be solved as a second order equation for $e^{\tau b_{i}}$, with two solutions. However, one is unacceptable, as it would lead to a negative value for $e^{\tau b_{i}}$. The positive solution of (11) then leads us to the decision level condition:

(12) $$b_{i}=\frac{1}{\tau }\log\left(\frac{\tau \Phi \lambda \left(l_{i}-l_{i-1}\right)e^{\tau y_{i-1}}}{2}+\frac{1}{2}\sqrt{4e^{\tau (y_{i-1}+y_{i})}+\tau^{2}\Phi^{2}\lambda^{2}{\left(l_{i}-l_{i-1}\right)}^{2}e^{2\tau y_{i-1}}}\right),$$

Finally, for codeword lengths, we have the well-known entropy condition:

(13) $$l_{i}=-\log_{2}\int_{b_{i}}^{b_{i+1}}f_{X}\left(x\right)dx,$$

**Figure 2:**
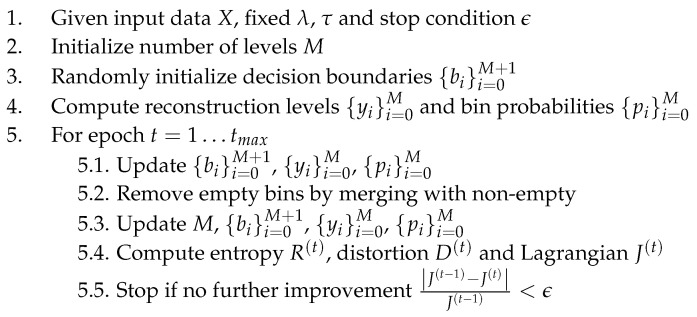
An iterative algorithm for entropy-constrained scalar $L_{\infty }$-oriented quantization.

Conditions (9), (12), and (13) are now sufficient to fully define $L_{\infty }$-oriented entropy-constrained scalar quantizers, the iterative design scheme being illustrated in Figure 2. The decision boundaries are initialized randomly around a fine uniform grid corresponding to a quantizer with $D_{\max}=1$, providing a dense set of closely spaced levels that ensure high initial resolution. For a fixed Lagrange multiplier λ, the optimization minimizes J=D+λR, producing a single operating point on the rate–distortion curve. By iteratively adjusting λ, the algorithm converges to the quantizer that satisfies a specified rate constraint (or the dual problem, distortion constraint).

Unlike uniform quantizers with fixed spacing and midpoint reconstruction levels, the proposed method updates boundaries and reconstruction values independently, producing nonuniform bins and non-midpoint reconstruction levels.

The computational complexity of this iterative scheme is governed by the sample–bin assignment and update operations. For *N* input samples, *M* quantization levels, and $t_{\max}$ iterations, the total cost scales as $O(t_{\max}\left(N+M\right))$, comparable to classical Lloyd–Max and entropy-constrained quantizer designs under a linear sweep implementation. The additional entropy and differentiable $L_{\infty }$ terms contribute only constant-factor overhead without affecting the asymptotic order.

It should be noted that (9), derived under the high-rate assumption, provides optimal reconstruction levels for continuous source distributions. However, for sparse source distributions, or at low rates, the variation of $f_{X}\left(x\right)$ within a bin can be significant, and the midpoint may no longer minimize the $L_{\infty }$ distortion. Consequently, we generalize (9) by defining the reconstruction level as the midpoint of the data points within each bin:

(14) $$y_{i}=\frac{\max(x)+\min(x)}{2},\;b_{i}\leq x<b_{i+1}.$$

It is obvious that for high-rate and continuous distributions, (14) reduces to the reconstruction levels given by (9).

Finally, since $D=\frac{1}{\tau }\log\Phi$, in practice, we compute Φ from *D* rather than using (6). At each iteration *t*, $\Phi^{\left(t\right)}$ is evaluated using $y_{i}^{\left(t-1\right)}$ and $b_{i}^{\left(t-1\right)}$ from the previous iteration, i.e., based on $D^{\left(t-1\right)}$. This procedure yields near-optimal solutions for ${\left\{b_{i}\right\}}_{i=0}^{M+1}$ and ${\left\{y_{i}\right\}}_{i=0}^{M}$, with the algorithm experimentally found to converge typically within 10–20 iterations.

## 4. Experimental Results

This section evaluates the rate-distortion performance of the proposed scalar $L_{\infty }$ quantizers under two representative scenarios: (i) memoryless sources following parametric distributions commonly used to model coding residuals or non-Gaussian stochastic processes, and (ii) training data with sparse or discontinuous distributions. In both cases, we benchmark the proposed approach against families of uniform quantizers.

In addition, we present a near-lossless $L_{\infty }$-bounded compression scheme for depth map video coding, wherein we equip the codec of [24] with the designed non-uniform $L_{\infty }$ quantizers. We compare its performance both with its uniform-quantizer baseline and with other state-of-the-art near-lossless coding methods.

### 4.1. Continuous and Discrete Parametric Distributions

We consider memoryless sources whose probability density functions belong to distribution families known to effectively model coding residuals or capture non-Gaussian stochastic behavior. In particular, we examine the TSGD distribution, respectively, the Laplacian and Exponential distributions. For each distribution, N=50,000 samples are generated using σ=10 (Laplacian), θ=0.9 (TSGD), and λ=10 (Exponential).

**Figure 3:**
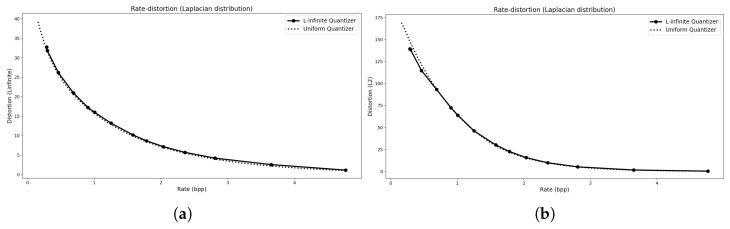
For a Laplacian distribution. (**a**) The R–D convex hull computed using the $L_{\infty }$ distortion metric. (**b**) The R–D curve showing the corresponding $L_{2}$ distortion values evaluated at the same operating points.

**Figure 4:**
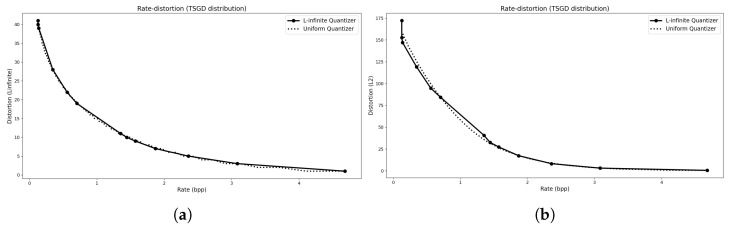
For a TSGD distribution. (**a**) The R–D convex hull computed using the $L_{\infty }$ distortion metric. (**b**) The R–D curve showing the corresponding $L_{2}$ distortion values evaluated at the same operating points.

**Figure 5:**
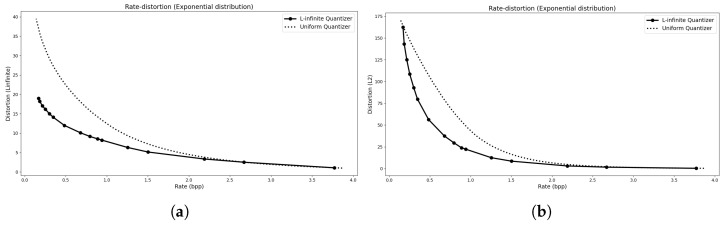
For an Exponential distribution. (**a**) The R–D convex hull computed using the $L_{\infty }$ distortion metric. (**b**) The R–D curve showing the corresponding $L_{2}$ distortion values evaluated at the same operating points.

The rate–distortion (R–D) curves for these distributions are shown in Figure 3, Figure 4 and Figure 5. In each case, we compare the convex hull of the proposed $L_{\infty }$ quantizer with that of a mid-tread deadzone uniform quantizer, where the rate is measured as entropy and the distortion corresponds to the empirical $L_{\infty }$ error, excluding any transmission overhead. The $L_{2}$ R–D curve for the same operating points is also shown for reference. The results indicate that the $L_{\infty }$ quantizers achieve essentially the same performance as their uniform counterparts for symmetric distributions (Laplacian and TSGD), while outperforming them for the asymmetric Exponential case.

The parameter settings for the R–D points are listed in Table 1, including the rate *R*, distortion *D*, initial and final number of levels $\left(M_{i},M_{f}\right)$, and the corresponding Lagrange multiplier λ. Each convex hull comprises 50 optimization runs with different λ values, using $\tau =\frac{\log N}{0.2}$. Larger τ values led to numerical instability, whereas reducing τ below $\frac{\log N}{4}$ caused minor deviations between theoretical and empirical results.

**Figure 6:**
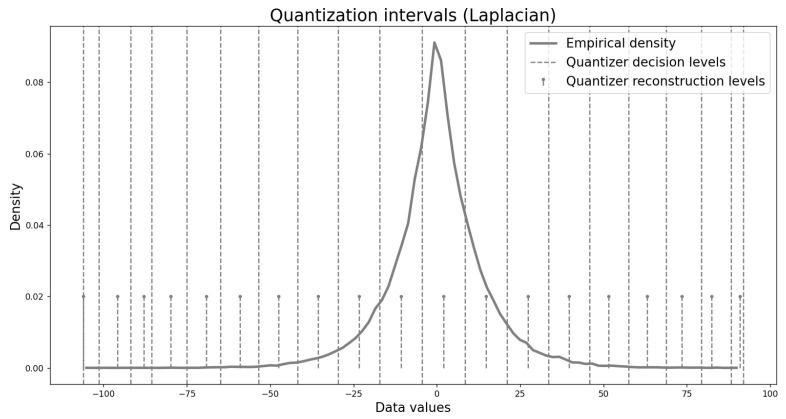
Quantization intervals for a Laplacian signal at distortion $D_{max}=5$.

An example of the resulting quantization intervals for a Laplacian source under an $L_{\infty }$ distortion bound of $D_{max}=5$ is shown in Figure 6. Apart from minor deviations at the edges (which can be adjusted without affecting the error), the resulting intervals coincide with those of a uniform quantizer. For the Exponential distribution, the intervals are nearly uniform but incorporate a shifted anchor point that accounts for the distribution’s asymmetry. This outcome is expected: the central reconstruction level of the proposed quantizer naturally aligns with an R–D optimal point, whereas for asymmetric distributions without a natural deadzone, uniform quantizers default to being anchored at zero unless prior knowledge of the source statistics is used. A uniform quantizer could, in principle, achieve comparable performance, but only if its anchor point were chosen according to such prior knowledge (e.g., mean, mode, or another relevant measure).

### 4.2. Sparse Source Distributions

**Figure 7:**
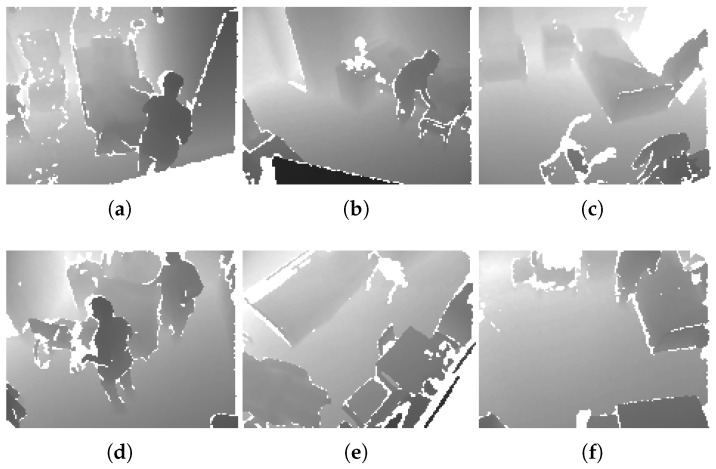
A sample depth map frame for dataset (**a**) S1, (**b**) S2, (**c**) S3, (**d**) S4, (**e**) S5, (**f**) S6.

We next evaluate the performance of non-uniform $L_{\infty }$ quantizers on a set of medical surveillance depth map video datasets. The data was captured with an Orbbec Persee depth camera in hospital room environments and consists of 16-bit depth frames and associated 8-bit segmentation maps. Part of this dataset has also been reported in prior work [24]. Representative frames from the sequences are shown in Figure 7, with datasets S2–S5 corresponding to Rooms 2–5 as used in [24].

In the following, we propose a modified encoder for the near-lossless $L_{\infty }$-bounded mode of the residual-based codec described in [24], which extends the original design by introducing non-uniform scalar $L_{\infty }$-optimized quantizers in place of the piecewise-uniform ones used previously.

For completeness, we summarize the architecture of this codec, which forms the foundation of our system. The codec performs intra-frame compression of depth video data using two inputs for each frame: the 16-bit depth frame itself and a semantic segmentation map, which is derived using machine-learning-based classifiers and associated with the same frame. In the first stage, each frame is separated into foreground and background regions based on the segmentation map. A reference background is then constructed from a set of static frames captured at the beginning of the encoding process and is updated whenever the camera position changes—specifically, when movement is detected through variations in segmentation labels within the reference background. Each incoming frame is subsequently subtracted from the reference background to obtain a residual image, while the foreground regions are preserved in lossless form. The residual is next processed by an $L_{\infty }$-oriented quantization block that maps residual values into quantization bins with a guaranteed per-pixel error bound. Finally, the quantized residual and foreground data are losslessly and independently encoded using JPEG-LS, while the reconstruction levels of the quantizer and other frame-level metadata are compressed using the Zlib library [39], producing compact frame packets that contain both the encoded data and all associated header information.

It should be noted that a separate quantizer is designed for each frame to adapt to temporal variations in the residual statistics. While sharing a single quantizer across multiple frames could slightly reduce signaling overhead and latency, the per-frame design offers superior adaptability with negligible additional header cost.

In order to compare quantizer performance, we first report coding results for the quantized residuals from these datasets. The corresponding reconstruction levels are included within the bitstream as compact header metadata, whose size is negligible compared to the frame payload. Therefore, Table 2 reports only the rate of the quantized residual data, isolating the impact of the quantizer design itself. Table 2 summarizes the average quantization rate (in bpp) for a given guaranteed $L_{\infty }$ distortion $D_{max}$. Results are shown for the codec equipped with our proposed non-uniform $L_{\infty }$-oriented quantizers, the piecewise uniform quantizers introduced in [24], and standard uniform quantizers. Across nearly all datasets and distortion levels, the proposed non-uniform quantizers achieve lower rates than both the uniform and piecewise-uniform approaches, demonstrating their superior efficiency, with only minor exceptions at extreme settings.

Table 3 further presents coding results for the complete video streams, showing the average bitrate per frame (in bpp), the average PSNR per frame (in dB), and the guaranteed $L_{\infty }$ distortion $D_{max}$. We compare the codec using non-uniform, piecewise-uniform, and standard uniform quantizers and also include results for JPEG-LS and CALIC. Overall, the codec equipped with our proposed $L_{\infty }$ non-uniform quantizers achieves the lowest bitrate across all datasets for nearly any given $D_{max}$. In terms of PSNR, it remains competitive and surpasses all designs at very high rates, while at low to medium rates, the uniform and piecewise-uniform quantizers yield superior PSNR values.

**Figure 8:**
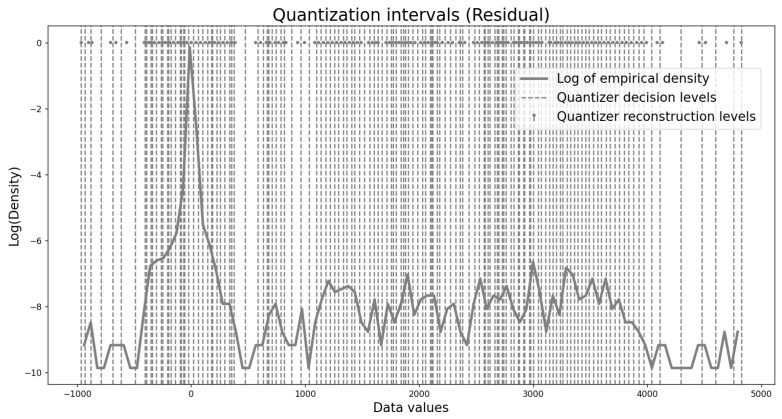
Quantization intervals for a depth map residual at distortion $D_{max}=20$.

Finally, to better understand how the proposed non-uniform quantizers adapt to sparse source distributions and to compare their behavior with the near-uniform quantizers obtained for the continuous Laplacian distribution in Figure 6, we visualize one example of the resulting quantization intervals. Figure 8 shows the intervals for a depth map residual at a distortion level of $D_{max}=20$. The residual distribution is extremely sparse, with a sharp peak and long tails. To reveal details in the tails, we plot the logarithm of the density rather than the density itself. The figure highlights that the resulting quantizers are highly non-uniform, in contrast to the near-uniform quantizers of the Laplacian case, demonstrating the ability of our design algorithm to adapt to sparse source distributions.

## 5. Discussion

The experimental results demonstrate several key aspects of the proposed $L_{\infty }$ quantizers. For parametric distributions such as Laplacian, TSGD, and Exponential sources, the design algorithm converges to nearly uniform quantizers. This is consistent with theory: when the distribution is continuous, unimodal, monotone, and without heavy sparsity, uniform binning is deemed $L_{\infty }$-optimal. Small deviations at the edges, as seen for the Laplacian case, have negligible impact on rate–distortion performance but confirm the algorithm’s ability to adapt to local distribution features. For asymmetric sources like the Exponential, the anchor point is shifted automatically, improving efficiency without altering the overall near-uniform structure.

In contrast, for sparse or discontinuous sources such as residuals in medical depth map sequences, the proposed non-uniform quantizers exhibit a highly non-uniform structure, reflecting an optimal allocation of quantization levels to minimize the $L_{\infty }$ error. This adaptive behavior directly translates into improved compression efficiency, as evidenced by the lower rates reported in Table 2 and Table 3.

The coding experiments further confirm that these benefits carry over to practical video compression. Integrated into a residual-based near-lossless $L_{\infty }$-constrained coding scheme, the proposed quantizers consistently yield lower bitrates than both uniform and piecewise-uniform designs, as well as standard near-lossless codecs like JPEG-LS and CALIC. On average, we observe gains of 43.9% over JPEG-LS, 19.5% over CALIC, 9.4% over standard uniform quantizers, and 2.9% over piecewise-uniform quantizers. In terms of PSNR, uniform quantizers perform better at low to medium rates, while the proposed $L_{\infty }$ designs match or surpass them at high rates (small $D_{\max}$) once sufficient levels ensure both maximum-error control and average fidelity. Overall, these results demonstrate that $L_{\infty }$ non-uniform quantization provides a powerful tool for applications requiring strict error guarantees alongside efficient coding of sparse or structured residual data.

## 6. Conclusions

In this paper, we presented a design framework for non-uniform $L_{\infty }$-oriented quantizers and evaluated their performance on both synthetic parametric sources and medical depth map datasets. We showed that while continuous, smoothly decaying distributions yield near-uniform quantizers, sparse or irregular sources benefit greatly from non-uniform bin allocation. Experimental results confirm that this adaptability not only improves rate–distortion performance over uniform and piecewise-uniform designs but also translates into significant bitrate savings when integrated into a residual-based near-lossless compression pipeline, surpassing state-of-the-art near-lossless schemes.

Overall, our results establish non-uniform $L_{\infty }$-oriented quantization as an effective approach for combining strict error control with improved compression efficiency, particularly for sparse or irregular data sources.

## Figures and Tables

**Table 1:** Summary of parameter configurations corresponding to points on the R–D convex hulls of the Laplacian, TSGD, and Exponential models. For each point, the rate *R*, distortion *D*, the initial number of quantization levels $M_{i}$, the final number of levels $M_{f}$ after convergence, and the associated Lagrangian multipliers λ are reported.

Laplacian					TSGD					Exponential				
R	D	$M_{i}$	$M_{f}$	λ	R	D	$M_{i}$	$M_{f}$	λ	R	D	$M_{i}$	$M_{f}$	λ
0.29	32.72	99	4	35.1	0.11	41.00	91	3	45.9	0.17	19.00	49	3	19.0
0.30	31.84	99	5	34.3	0.12	40.00	91	3	43.9	0.18	18.19	49	3	18.2
0.46	26.19	99	6	27.8	0.14	39.00	91	3	42.9	0.22	17.04	49	4	17.1
0.69	20.97	99	7	21.2	0.34	28.00	91	5	30.6	0.25	16.17	49	4	15.9
0.91	17.24	99	9	17.1	0.56	22.00	91	6	23.5	0.30	15.00	49	4	14.7
1.01	15.98	99	8	15.5	0.70	19.00	91	7	20.4	0.35	14.11	49	4	13.6
1.25	13.19	99	10	12.2	1.35	11.00	91	10	11.2	0.48	12.01	49	5	11.2
1.58	10.20	99	12	9.0	1.44	10.00	91	10	10.2	0.68	10.11	49	6	8.9
1.78	8.64	99	15	7.3	1.57	9.00	91	10	9.2	0.79	9.16	49	7	7.8
2.04	7.16	99	18	5.7	1.87	7.00	91	14	7.1	0.89	8.54	49	7	7.0
2.36	5.69	99	22	4.1	2.36	5.00	91	21	4.1	0.94	8.21	49	7	6.6
2.81	4.21	99	31	2.4	3.08	3.00	91	34	2.0	1.25	6.31	49	10	4.7
3.65	2.55	99	50	0.8	4.68	1.00	91	86	1.0	1.50	5.14	49	12	3.5
4.77	1.14	99	88	0.5						2.19	3.34	49	20	1.6
										2.67	2.51	49	25	0.8
										3.77	1.07	49	46	0.5

**Table 2:** Comparison of near-lossless compression results for residual frames, for different schemes. Rates (in bpp) are shown, together with guaranteed maximum distortion values $D_{max}$.

Data	$D_{\max}$	Codec w/Quant.		
		Non-Unif.	pw-Unif.	Unif.
S1	1	**2.819**	2.835	3.685
	10	**2.287**	2.334	2.639
	20	**1.965**	2.062	2.216
	30	**1.610**	1.665	1.769
S2	1	**2.335**	2.386	2.968
	10	**1.792**	1.794	2.043
	20	**1.505**	1.632	1.807
	30	**1.278**	1.370	1.491
S3	1	**2.084**	2.093	2.608
	10	**1.611**	1.634	1.818
	20	**1.265**	1.419	1.538
	30	**1.053**	1.092	1.152
S4	1	**2.584**	2.616	3.311
	10	**2.018**	2.023	2.280
	20	**1.608**	1.796	1.963
	30	1.318	**1.257**	1.343
S5	1	**2.614**	2.635	3.248
	10	**1.989**	2.023	2.241
	20	**1.648**	1.688	1.801
	30	**1.316**	1.374	1.446
S6	1	**2.663**	2.688	3.330
	10	**2.039**	2.093	2.322
	20	**1.707**	1.800	1.937
	30	1.422	**1.408**	1.506

*Note.* Bold values indicate the best rate obtained for each dataset and $D_{max}$.

**Table 3:** Comparison of near-lossless compression results for different schemes. Rates (in bpp) and PSNR (dB) values are shown, together with guaranteed maximum distortion values $D_{max}$.

Data	$D_{\max}$	JPEG-LS		CALIC		Codec w/Non-Unif.		Codec w/pw-Unif.		Codec w/Unif.	
		Rate	PSNR	Rate	PSNR	Rate	PSNR	Rate	PSNR	Rate	PSNR
S1	1	7.119	99.99	4.712	100.27	**3.395**	120.43	3.471	119.62	4.135	119.62
	10	4.826	65.05	3.142	65.94	**2.833**	61.49	2.877	82.20	3.088	82.20
	20	3.990	53.80	2.593	54.93	**2.497**	57.26	2.591	70.43	2.666	70.43
	30	3.459	47.26	3.332	48.42	**2.128**	49.10	2.182	61.55	2.219	61.55
S2	1	6.302	99.97	4.611	100.16	**2.875**	120.47	2.975	119.11	3.389	119.11
	10	3.994	65.02	3.089	65.84	2.300	71.42	**2.297**	84.77	2.464	84.77
	20	3.215	53.13	2.529	54.44	**2.001**	53.13	2.120	73.89	2.228	73.89
	30	2.774	46.76	2.240	48.00	**1.764**	47.16	1.849	62.85	1.913	62.85
S3	1	5.540	100.11	3.919	101.19	**2.291**	122.33	2.356	120.55	2.709	120.55
	10	3.653	65.41	2.564	66.73	**1.794**	59.94	1.815	84.47	1.920	84.47
	20	2.800	53.80	2.091	56.00	**1.435**	51.55	1.587	73.82	1.640	73.82
	30	2.301	47.19	1.855	49.12	**1.212**	49.85	1.248	61.80	1.254	61.80
S4	1	6.320	99.96	4.192	100.05	**2.975**	119.91	3.070	119.17	3.587	119.17
	10	3.978	65.00	2.791	65.50	**2.377**	64.82	2.381	82.71	2.556	82.71
	20	3.300	53.65	2.270	54.29	**1.954**	55.34	2.140	68.75	2.238	68.75
	30	2.876	47.05	2.026	47.79	1.653	49.20	**1.588**	58.93	1.618	58.93
S5	1	6.722	100.05	4.265	100.79	**2.782**	120.57	2.860	119.30	3.298	119.30
	10	4.655	65.38	2.872	66.06	**2.130**	64.61	2.160	86.31	2.291	86.31
	20	3.892	53.78	2.364	55.00	**1.775**	57.87	1.811	71.12	1.851	71.12
	30	3.360	47.08	2.111	48.59	**1.430**	52.23	1.484	61.45	1.496	61.45
S6	1	5.841	100.05	3.932	100.33	**3.308**	118.97	3.425	117.52	3.832	117.52
	10	3.764	65.30	2.592	65.94	**2.631**	60.59	2.687	82.59	2.825	82.59
	20	2.978	53.57	2.089	54.37	**2.281**	56.26	2.374	68.23	2.439	68.23
	30	2.500	46.91	1.860	48.09	1.983	49.08	**1.967**	59.78	2.008	59.78

*Note.* Bold values indicate the best rate obtained for each dataset and $D_{max}$.

## Data Availability

Restrictions apply to the availability of this data. Data was obtained from Mintt S.A., Belgium and is available from the authors with the permission of Mintt S.A., Belgium.

## Appendix A. Proof and Derivation Details

In the following, we give detailed proof of Theorem 1; respectively, derivation of Equation (5) up to (13).

**Proof** **of** **Theorem** **1.** Let us define $U=\tau \max_{j=1\ldots N}\left|Z_{j}\right|$ with some parameter τ>0 and where $Z_{j}=X_{j}-Q\left(X_{j}\right)$. By Jensen’s inequality φ(E[U])≤E[φ(U)] for a convex function φ, it follows that:

(A1) $$e^{\tau E\left[\max_{j}\left|Z_{j}\right|\right]}\leq E\left[e^{\tau \max_{j}\left|Z_{j}\right|}\right]\leq E\left[\sum_{j=1}^{N}e^{\tau |Z_{j}|}\right]\leq NE\left[e^{\tau \max_{j}\left|Z_{j}\right|}\right],$$

Taking the logarithm in (A1) and using Jensen again, we obtain:

(A2) $$\begin{array}{c} \log e^{\tau E\left[\max_{j}\left|Z_{j}\right|\right]}=\tau E\left[\max_{j}\left|Z_{j}\right|\right]\leq \log E\left[\sum_{j=1}^{N}e^{\tau |Z_{j}|}\right]\leq \log N+\log E\left[e^{\tau \max_{j}\left|Z_{j}\right|}\right]\\ \leq \log N+E\left[\log e^{\tau \max_{j}\left|Z_{j}\right|}\right]=\log N+\tau E\left[\max_{j}\left|Z_{j}\right|\right] \end{array}$$

Further dividing by τ leads to:

(A3) $$E\left[\max_{j}\left|Z_{j}\right|\right]\leq \frac{1}{\tau }\log E\left[\sum_{j=1}^{N}e^{\tau |Z_{j}|}\right]\leq E\left[\max_{j}\left|Z_{j}\right|\right]+\frac{\log N}{\tau },$$

It is interesting to observe that Equation (A3) describes in fact the well-known bounds of the LogSumExp function $\log\sum_{j=1}^{N}e^{\tau |Z_{j}|}$. Furthermore, we can write this in terms of a single random variable as:

(A4) $$E\left[\sum_{j=1}^{N}e^{\tau |Z_{j}|}\right]=NE\left[e^{\tau |Z|}\right],$$

The middle term can be written in terms of a single random variable as $E\left[\sum_{j=1}^{N}e^{\tau |Z_{j}|}\right]=NE\left[e^{\tau |Z|}\right]$, such that (A3) leads to:

(A5) $$\frac{1}{\tau }\log E\left[e^{\tau |Z|}\right]\leq E\left[\max_{j}\left|Z_{j}\right|\right]\leq \frac{1}{\tau }\log E\left[e^{\tau |Z|}\right]+\frac{\log N}{\tau },$$

Using the above relation, Equation (A3) becomes:

(A6) $$E\left[\max_{j}\left|Z_{j}\right|\right]\leq \frac{1}{\tau }\log NE\left[e^{\tau |Z|}\right]=\frac{\log N}{\tau }+\frac{1}{\tau }\log E\left[e^{\tau |Z|}\right]\leq \frac{\log N}{\tau }+E\left[\max_{j}\left|Z_{j}\right|\right],$$

Re-arranging the terms, we obtain:

(A7) $$\frac{1}{\tau }\log E\left[e^{\tau |Z|}\right]\leq E\left[\max_{j}\left|Z_{j}\right|\right]\leq \frac{1}{\tau }\log E\left[e^{\tau |Z|}\right]+\frac{\log N}{\tau },$$

Next, for the quantization error (distortion), we have the well-known generalized relation:

(A8) $$D\left(Q\right)=E\left[d(X,Q(X))\right]=\int_{-\infty }^{\infty }d\left(x,Q\left(x\right)\right)f_{X}\left(x\right)dx=\sum_{i=0}^{M}\int_{b_{i}}^{b_{i+1}}d\left(x,y_{i}\right)f_{X}\left(x\right)dx,$$

Replacing the $L_{\infty }$ quantization error approximation in Equation (A8) leads to:

(A9) $$\begin{array}{c} E\left[d(X,Q(X))\right]\approx \frac{1}{\tau }\log E\left[e^{\tau |Z|}\right]=\frac{1}{\tau }\log E\left[e^{\tau |X-Q(X)|}\right]\\ =\frac{1}{\tau }\log\sum_{i=0}^{M}\int_{b_{i}}^{b_{i+1}}e^{\tau |x-y_{i}|}f_{X}\left(x\right)dx, \end{array}$$

Which gives us Equation (1) and concludes the proof. □

Let us start from the Lagrangian formulation for $L_{\infty }$-oriented quantization as given by Equation (5):

(A10) $$J=\frac{1}{\tau }\log\sum_{i=0}^{M}\int_{b_{i}}^{b_{i+1}}e^{\tau |x-y_{i}|}f_{X}\left(x\right)dx+\lambda \sum_{i=0}^{M}l_{i}\int_{b_{i}}^{b_{i+1}}f_{X}\left(x\right)dx,$$

We wish to find the optimal reconstruction levels ${\left\{y_{i}\right\}}_{i=0}^{M}$ and decision levels ${\left\{b_{i}\right\}}_{i=0}^{M+1}$ that minimize (A10). For high rates, we assume that the density function $f_{X}\left(x\right)$ is constant over each quantization interval $\left[b_{i},b_{i+1}\right)$, i.e., for each $y_{i}\in \left[b_{i},b_{i+1}\right)$ has some constant value $f_{X}\left(y_{i}\right)=\ldots =f_{X}\left(b_{i}\right)$. Also, we define Φ as:

(A11) $$\Phi =\sum_{i=0}^{M}\int_{b_{i}}^{b_{i+1}}e^{\tau |x-y_{i}|}f_{X}\left(x\right)dx,$$

In a first instance, we compute $\frac{dJ}{dy_{i}}$. Recall that for any function *u*, the derivative of the logarithm is given by ${\left(\log u\right)}^{\prime }=\frac{u^{\prime }}{u}$. Since the second term of Equation (A10) does not depend on $y_{i}$, and knowing that $b_{i}\leq y_{i}<b_{i+1}$, the derivative can then be written as:

(A12) $$\begin{array}{c} \frac{dJ}{dy_{i}}=\frac{f_{X}\left(y_{i}\right)}{\tau \Phi }\frac{d}{dy_{i}}\left(\int_{y_{i}}^{b_{i+1}}e^{\tau (x-y_{i})}dx+\int_{b_{i}}^{y_{i}}e^{\tau (y_{i}-x)}dx\right)\\ =\frac{f_{X}\left(y_{i}\right)}{\tau \Phi }\frac{d}{dy_{i}}\left(\frac{e^{\tau (b_{i+1}-y_{i})}-1}{\tau }+\frac{-1+e^{\tau (y_{i}-b_{i})}}{\tau }\right)\\ =\frac{f_{X}\left(y_{i}\right)}{\tau \Phi }\left(-e^{\tau (b_{i+1}-y_{i})}+e^{\tau (y_{i}-b_{i})}\right), \end{array}$$

Setting the above expression to zero leads us to the following equation:

(A13) $$e^{\tau (y_{i}-b_{i})}-e^{\tau (b_{i+1}-y_{i})}=0,$$

From (A13), it is now trivial to compute the reconstruction level condition:

(A14) $$y_{i}=\frac{b_{i}+b_{i+1}}{2},$$

We now wish to compute $\frac{dJ}{db_{i}}$. First, recall the Leibniz integral rule for some function g(x,t) and some integration limits a(x),c(x):

(A15) $$\frac{d}{dx}\int_{a(x)}^{c(x)}g\left(x,t\right)dt=g\left(x,c\left(x\right)\right)\frac{dc(x)}{dx}-g\left(x,a\left(x\right)\right)\frac{da(x)}{dx}+\int_{a(x)}^{c(x)}\frac{\partial g(x,t)}{\partial x}dt,$$

which for the special case a(x)=a and c(x)=x can be written as:

(A16) $$\frac{d}{dx}\int_{a}^{x}g\left(x,t\right)dt=g\left(x,x\right)+\int_{a}^{x}\frac{\partial g(x,t)}{\partial x}dt,$$

Starting from Equation (A10), we can now compute $\frac{dJ}{db_{i}}$ by identifying the terms that depend on $b_{i}$, respectively, using the fact that the derivative ${\left(\log u\right)}^{\prime }=\frac{u^{\prime }}{u}$ for any function *u*. We thus obtain the following equation, where we have also flipped the integral limits (hence the negative signs) in order to match the special case expression of the Leibniz rule:

(A17) $$\begin{array}{c} \frac{dJ}{db_{i}}=\frac{1}{\tau \Phi }\frac{d}{db_{i}}\left(-\int_{b_{i+1}}^{b_{i}}e^{\tau |x-y_{i}|}f_{X}\left(x\right)dx+\int_{b_{i-1}}^{b_{i}}e^{\tau |x-y_{i-1}|}f_{X}\left(x\right)dx\right)\\ +\lambda \frac{d}{db_{i}}\left(-l_{i}\int_{b_{i+1}}^{b_{i}}f_{X}\left(x\right)dx+l_{i-1}\int_{b_{i-1}}^{b_{i}}f_{X}\left(x\right)dx\right), \end{array}$$

Applying the Leibniz special case rule to the above expression gives us the following:

(A18) $$\frac{dJ}{db_{i}}=\frac{1}{\tau \Phi }\left(-e^{\tau |b_{i}-y_{i}|}f_{X}\left(b_{i}\right)+e^{\tau |b_{i}-y_{i-1}|}f_{X}\left(b_{i}\right)\right)+\lambda \left(-l_{i}f_{X}\left(b_{i}\right)+l_{i-1}f_{X}\left(b_{i}\right)\right),$$

where the second terms $\int_{a}^{x}\frac{\partial g(x,t)}{\partial x}dt$ of the Leibniz rule are always zero since in our case g(x,t) does not depend on $b_{i}$. Given that $y_{i-1}<b_{i}\leq y_{i}$, the above expression now simplifies to:

(A19) $$\frac{dJ}{db_{i}}=f_{X}\left(b_{i}\right)\left(\frac{e^{\tau (b_{i}-y_{i-1})}-e^{\tau (y_{i}-b_{i})}}{\tau \Phi }-\lambda \left(l_{i}-l_{i-1}\right)\right),$$

Setting Equation (A19) to zero results in the following equation for $e^{\tau b_{i}}$:

(A20) $$e^{2\tau b_{i}}-\tau \Phi \lambda \left(l_{i}-l_{i-1}\right)e^{\tau y_{i-1}}e^{\tau b_{i}}-e^{\tau (y_{i-1}+y_{i})}=0,$$

Equation (A20) can be solved as a second order equation in $e^{\tau b_{i}}$, with the following solutions:

(A21) $$e^{\tau b_{i}}=\frac{\tau \Phi \lambda \left(l_{i}-l_{i-1}\right)e^{\tau y_{i-1}}\pm \sqrt{\tau^{2}\Phi^{2}\lambda^{2}{\left(l_{i}-l_{i-1}\right)}^{2}e^{2\tau y_{i-1}}+4e^{\tau (y_{i-1}+y_{i})}}}{2},$$

Since $4e^{\tau (y_{i-1}+y_{i})}>0$, it can be seen that one solution will be negative regardless of the sign of $\left(l_{i}-l_{i-1}\right)$, which is unacceptable, as we always have $e^{\tau b_{i}}>0$. Taking the positive solution and applying the logarithm leads us to the decision level condition:

(A22) $$b_{i}=\frac{1}{\tau }\log\left(\frac{\tau \Phi \lambda \left(l_{i}-l_{i-1}\right)e^{\tau y_{i-1}}}{2}+\frac{1}{2}\sqrt{4e^{\tau (y_{i-1}+y_{i})}+\tau^{2}\Phi^{2}\lambda^{2}{\left(l_{i}-l_{i-1}\right)}^{2}e^{2\tau y_{i-1}}}\right),$$

Finally, for codeword lengths, we have the well-known entropy condition:

(A23) $$l_{i}=-\log_{2}\int_{b_{i}}^{b_{i+1}}f_{X}\left(x\right)dx,$$

Conditions (A14), (A22), and (A23) now fully describe $L_{\infty }$-oriented entropy-constrained scalar quantizers, which concludes the proof.
